# Single-step hydrothermal synthesis of zinc oxide nanorods for potential use as nano-antibiotics without seeding or bases

**DOI:** 10.1371/journal.pone.0313224

**Published:** 2024-11-04

**Authors:** Chau Nguyen Minh Hoang, Khanh Duy Nguyen, Thuong Thi Ha Luong, Son Hai Nguyen, Mai Thi Tran

**Affiliations:** 1 VinUni-Illinois Smart Health Center, VinUniversity, Hanoi, Vietnam; 2 College of Health Science, VinUniversity, Hanoi, Vietnam; 3 School of Mechanical Engineering, Hanoi University of Science and Technology, Hanoi, Vietnam; 4 College of Engineering and Computer Science, VinUniversity, Hanoi, Vietnam; VIT University, INDIA

## Abstract

The alarming global rise in antibiotic resistance, driven by the widespread overuse of traditional antibiotics, has created an urgent demand for new antimicrobial solutions. This study presents zinc oxide (ZnO) nanorods as a potential nano-antibiotic agent. ZnO nanorods, with a 2:3 aspect ratio, were synthesized using an efficient one-step hydrothermal method at a low temperature of 100°C, reducing the synthesis time to just 5 hours. The synthesized ZnO nanorods’ morphology, structure, and composition were characterized using scanning electron microscopy, x-ray diffraction, and energy dispersive x-ray spectroscopy. The potent antimicrobial activity of these nanorods against common bacterial strains such as *Escherichia coli*, *Bacillus subtilis*, and *Vibrio parahaemolyticus* was examined through optical density at 600 nm (OD_600_) measurements and inhibition zone analysis, demonstrating substantial inhibition of bacterial growth. In particular, at a concentration of 5 mg/mL, ZnO nanorods achieved a 96% reduction of *B*. *subtilis* bacteria in OD_600_ and an impressive 99.87% reduction in culturing assays within one day, showcasing bactericidal efficiency on par with tetracycline at 0.003 mg/mL. Furthermore, a predictive model of bacterial growth was developed and validated, providing insights into the time-dependent bactericidal efficiency of the synthesized nanorods. These results highlight the potential of ZnO-based composites as a promising solution to combat antibiotic resistance, paving the way for next-generation antimicrobial materials.

## Introduction

Antibiotic resistance remains one of the most public health concerns worldwide, referring to the development of ineffectiveness against bacterial antibiotics [1]. Bacterial pathogens are constantly developing ways to resist antibiotics, making treatments that were once effective no longer viable [2]. Multiple mechanisms have been discovered, from the transfer of genetic material among organisms to mutations, that lead to bacterial modifications countering antibiotic effects, resulting in post-operative care complications, prolonged hospital stays, and treatment failures, further contributing to increased morbidity and mortality [3]. Being a challenge to all countries, antibiotic resistance is reported to be more prominent in low- and middle-income nations and is associated with excessive antibiotics without a medical prescription [1]. The environment also affects antibiotic resistance, as resistance development can occur in insufficiently treated sewage or hospital waste [4]. Hence, strategies to address antibiotic resistance must focus on appropriate antibiotic usage, tackling environmental factors, and seeking replacements for existing antibiotics.

To overcome antibiotic resistance, the scientific community is actively exploring alternative therapeutic methods. Phage therapy, employing bacteriophages to target and destroy bacteria, presents a particular approach, circumventing some of the broad-spectrum pitfalls of antibiotics [5]. However, phage therapy’s specificity can also be a disadvantage, as it requires precise bacterial strain identification, limiting its quick application in acute infections. Antimicrobial peptides offer another promising avenue, known for their fast action and lower propensity for resistance development [6]. Nevertheless, stability, toxicity, and cost issues hinder their widespread adoption. Additionally, the use of probiotics to maintain and restore healthy microbiomes shows promise in preventing infections. However, science is still evolving regarding the most effective strains and formulations [6]. Each method brings unique advantages and challenges, underscoring the need for further research and development to harness their full potential as replacements for traditional antibiotics.

In response to increasing antibiotic resistance and the urgent need for alternatives, recent literature increasingly emphasizes the importance of nanomaterials [7]. Nanomaterials offer distinct advantages over conventional antibiotics with their unique physical and chemical properties at the nanoscale. Their small sizes allow them to interact closely with microbial membranes, disrupting the structure and leading to cell death [8]. Moreover, nanomaterials’ high surface area-to-volume ratio allows for a more concentrated and effective interaction with bacterial cells. This increased reactivity also facilitates multiple modes of action, reducing the likelihood of resistance development. Additionally, nanomaterials can be engineered to target specific pathogens, thus limiting the impact on the beneficial microbiota and reducing side effects [7]. These attributes, combined with the possibility of functionalizing nanomaterials to enhance their specificity and biocompatibility, make them a focus of intense research as a promising solution to the growing antibiotic resistance problem [8].

Among nanomaterials, zinc oxide has emerged as one of the promising candidates for new antimicrobial agents. The interest in ZnO is attributed to its potent and broad-spectrum antimicrobial activity, which can combat various bacteria, fungi, and viruses [9–11]. Additionally, its biocompatibility makes it suitable for human use, reflected in its widespread inclusion in many consumer products [12]. With the scalable synthesis and cost-effective production, alongside its recognized safety profile, ZnO nanoparticles hold the potential to revolutionize the treatment of infections and reduce reliance on traditional antibiotics [12,13]. Thus, ZnO not only stands as a robust antimicrobial but also represents a stride toward sustainability in healthcare practices, ensuring its relevance and appeal in antibiotic research.

Nanomaterial properties such as size, shape, surface roughness, and energy significantly influence their antibacterial effects, primarily determined by the synthesis process [14,15]. For instance, zinc oxide nanorods (ZnO NRs) possess unique physicochemical properties that enhance their antimicrobial potential [16–18]. ZnO NRs are effective against a wide range of pathogens, including gram-positive bacteria like *B*. *subtilis* and *Staphylococcus aureus*, gram-negative bacteria such as *E*. *coli*, *Salmonella typhimurium*, *and Klebsiella pneumonia*, as well as fungi like *Aspergillus* [19,20]. Despite their effectiveness, the synthesis of ZnO NRs typically requires multiple steps and high temperatures, and their time-dependent inhibitory effects are underexplored [21,22]. In this study, we introduce a simplified, single-step hydrothermal method for synthesizing ZnO NRs. This method eliminates the traditional seeding and base-formation processes and operates at low temperatures compared to previous approaches that required higher hydrothermal temperatures over longer durations [23,24]. We evaluated the antimicrobial effectiveness of these nano-antibiotics against three microorganisms (*E*. *coli*, *B*. *subtilis*, and *V*. *parahaemolyticus)* and compared the antibacterial activity of ZnO at concentrations of 5, 10, and 50 mg/ml with that of tetracycline (TET) at 0.003 mg/mL over time. Our analysis provides insights into the antibacterial efficacy of ZnO NRs at different stages of bacterial growth, guiding optimized treatment strategies. This research aims to develop a new class of efficient, cost-effective nano-antibiotics that can be quickly produced at scale, addressing the urgent demand for novel antimicrobial agents in the face of rising antibiotic resistance.

## Experiments

### Chemicals and ZnO NRs preparation

All chemicals were used as received, including Zinc Acetate (Zn(CH₃COO)₂·2H₂O, 99.99%, Merck, Germany), Sodium Hydroxide (NaOH, 99%, Merck, Germany), Ethanol (C₂H₅OH, 99.5%, Xilong Scientific Co., Ltd., Guangdong, China), Tetracycline Hydrochloride (ultra-pure, C₂₂H₂₄N₂O₈·HCl, Bio Basic, Canada), and deionized water (DI).

**Fig 1:**
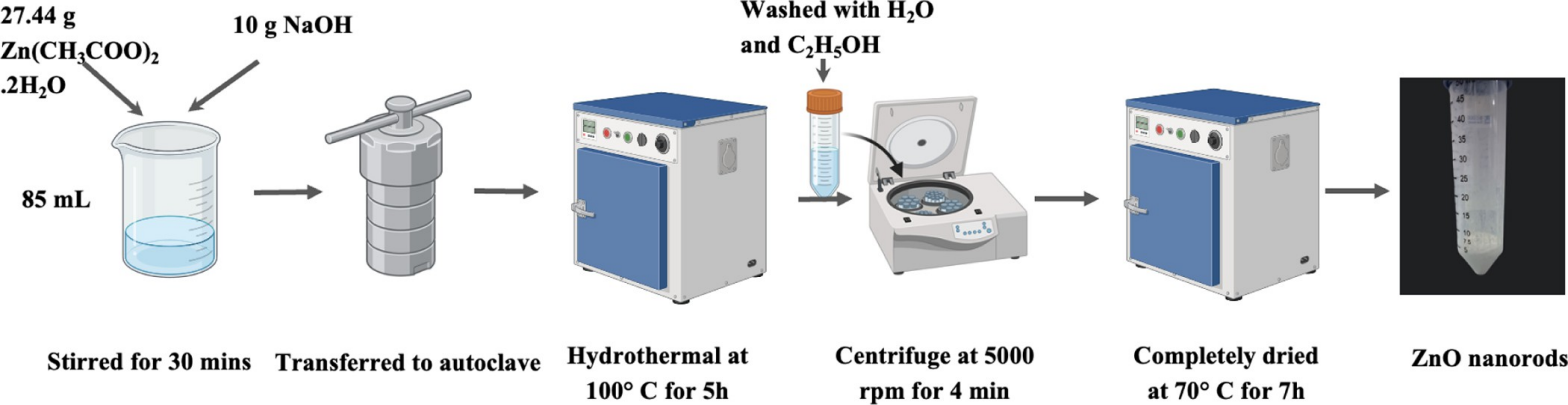
The schematic of ZnO nanomaterials preparation using the hydrothermal method.

The preparation of ZnO NRs is depicted in Fig 1. Briefly, 27.44 grams of Zn(CH₃COO)₂·2H₂O was dissolved in 67.5 mL of deionized water, and 10 grams of NaOH was dissolved in 17.5 mL of deionized water. The two solutions were then combined and stirred for 30 minutes. The mixture was transferred to a 200 mL stainless steel autoclave, sealed, and maintained at 100°C for 5 hours. After cooling to room temperature, the resultant white precipitate was isolated by centrifugation, washed three times with distilled water and ethanol to remove residue, and dried at 70°C for 7 hours.

### The procedure of bacterial inhibitory experiments

#### Bacterial growth conditions

*Escherichia coli*, *Bacillus subtilis*, and *Vibrio parahaemolyticus* were grown on nutrient medium Luria-Bertani medium (LB, Sigma-Aldrich) overnight at 37°C for growth assays.

#### Investigation of bacterial growth inhibition

The three concentrations of ZnO nanorods (5, 10, 50 mg/mL) were prepared by diluting the ZnO NRs stock solution with LB broth. A 10 mL bacterial suspension with an initial OD_600_ of 0.03 was incubated with 5, 10, 50 mg/mL of ZnO NRs in 15 mL Falcon tubes at 37°C. The bacterial culture without ZnO nanorods served as the negative control, while the culture treated with the antibiotic tetracycline (0.003 mg/mL) served as the positive control.

The turbidity of bacterial suspensions treated with ZnO NRs, along with the negative and positive controls, was measured OD_600_ using a DeNovix UV-Vis spectrometer at intervals of 0h, 2h, 4h, 6h, and 24h. The OD_600_ of the ZnO nanoparticle suspension at each concentration was measured and subtracted from the test reading to remove any interference from the nanoparticles. The percentage of bacterial growth inhibition after each exposure time to ZnO nanoparticles was calculated using the following equation:

$$Percentage\;of\;growth\;inhibition=\frac{OD\;control-OD\;test}{OD\;control}\times 100\%$$ (1)

#### Inhibition zone

After solidifying the nutrient agar in petri dish plates, 5 mm diameter wells were created in the middle of the agar. The bacteria were then evenly spread into the dish. ZnO nanorods were added to these wells at 5, 10, and 50 mg/ml concentrations. The plates were then incubated for 24 hours at 37°C. Subsequently, the diameter of the inhibition zone (in mm) formed around the well was measured to compare the effectiveness of different ZnO concentrations and antibiotics.

## Results

### Characterization of prepared ZnO NRs

**Fig 2:**
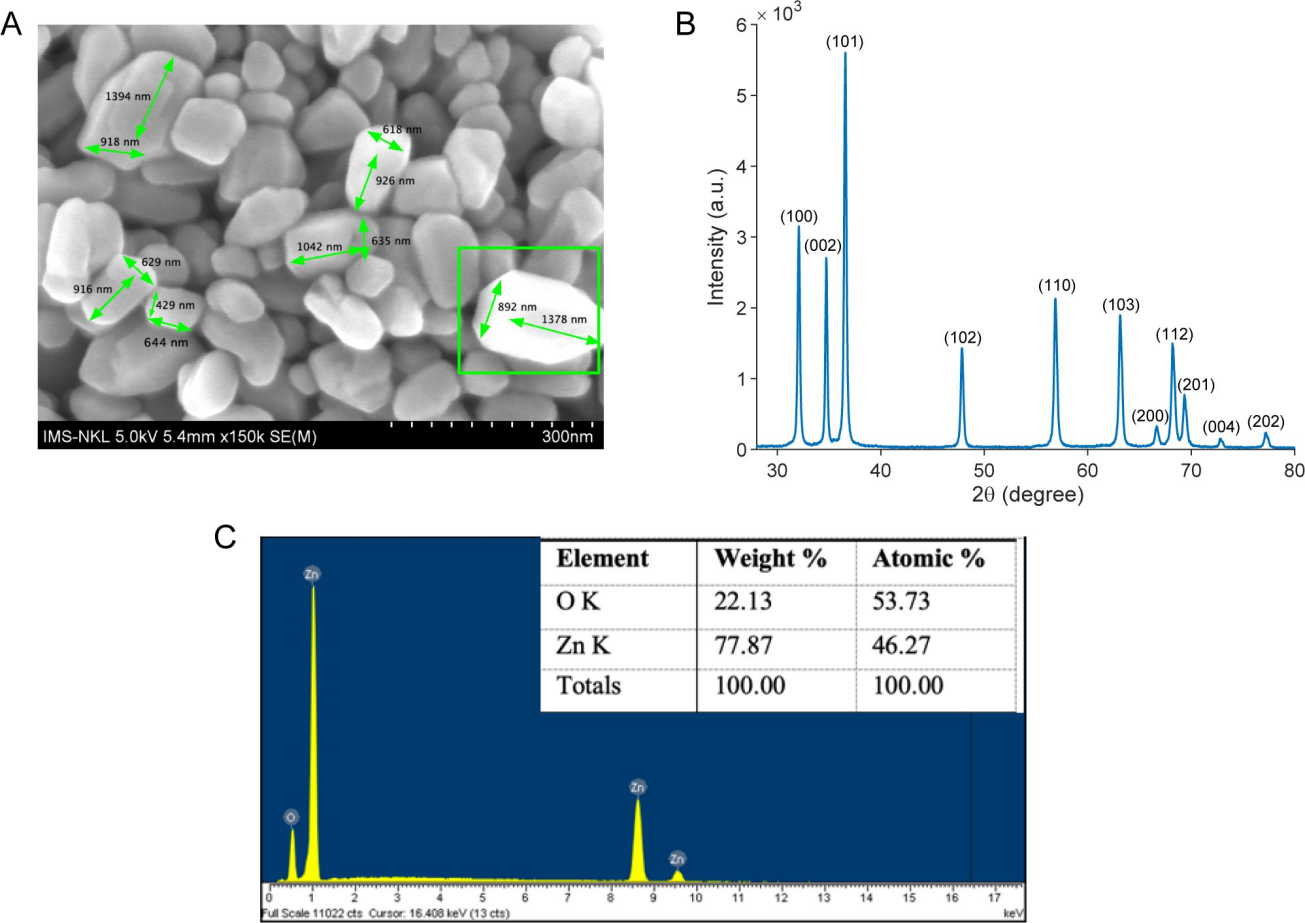
Characteristics of the prepared ZnO NRs. (A) The scanning electron microscopy (SEM) image of prepared materials taken by HITACHI-S4800. (B) The x-ray diffraction (XRD) patterns of prepared materials taken by Rigaku MiniFlex600. (C) The energy dispersive x-ray spectroscopy (EDX) image was taken by HITACHI S-4800 (Hitachi High-Tech, Japan).

We characterized the prepared materials using SEM, XRD, and EDX to investigate their morphology, structure, and composition. Fig 2A shows clearly defined nanorods with a hexagonal shape. Their dimensions vary, but the ratio between the hexagon’s diameter and length is roughly 2:3. This shape is consistent with the wurtzite hexagonal phase observed in the XRD pattern (Fig 2B). The XRD peaks appear at the (100), (002), (101), (102), (110), (103), (200), (112), (201), (004), and (202) planes, corresponding to 2θ angles of 32.08°, 34.74°, 36.56°, 47.86°, 56.86°, 63.16°, 66.74°, 68.24°, 69.36°, 73.18°, and 77.24°, respectively. All peaks correspond to the hexagonal ZnO structure with lattice constants a = 3.285 and c = 5.126 (JCPDS card no. 36–1451). The sharp and narrow diffraction peaks indicate high crystallinity, while the absence of additional peaks confirms the product’s purity. This purity is confirmed in the EDX spectrum (Fig 2C), which shows only Zn and O. Thus, we successfully synthesized ZnO nanorods through a straightforward one-step hydrothermal method at a low temperature of 100^ο^C in just 5 hours.

### Evaluating the bactericidal efficacy of ZnO nanorods: Utilizing OD_600_ measurements and inhibition zone analysis

**Fig 3:**
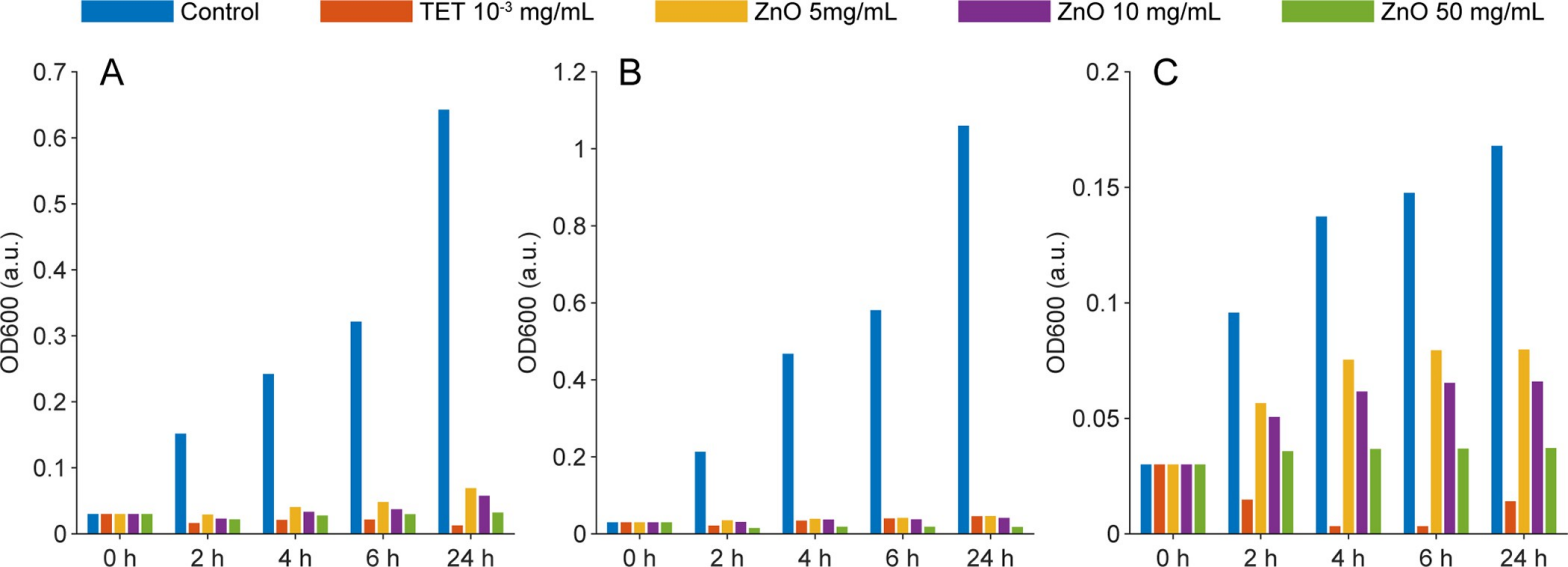
Antimicrobial activity of ZnO nanorods against. (A) *E*. *coli*, (B) *B*. *subtilis*, (C) *V*. *parahaemolyticus* measured by OD at 600 nm after incubation at 37°C, 150 rpm at different time points: 0h, 2h, 4h, 6h, and 24h.

In our exploration of ZnO nanorods as potential antibacterial agents, their antimicrobial activity was tested against Gram-positive (*B*. *subti*lis) and Gram-negative (*E*. *coli* and *V*. *parahaemolyticus)* bacteria at various concentrations (5, 10, 50 mg/mL) by measuring OD_600_. Results displayed in Fig 3 indicate that ZnO nanorods exert a more pronounced inhibitory effect on the growth of both *E*. *coli* (Fig 3A) and *B*. *subtilis* (Fig 3B) compared to *V*. *parahaemolyticus* (Fig 3C) across all concentrations over time. Based on the measured OD_600_ data in the S1 File, growth inhibition percentages were calculated using Eq (1) and summarized in Table 1. Notably, the inhibitory effect on *E*. *coli* and *B*. *subtilis* was nearly as potent as those of the positive control, tetracycline 0.003 mg/mL, at identical time points. For instance, ZnO concentrations of 5 and 10 mg/mL demonstrated similar inhibitory effects on *B*. *subtilis* as tetracycline. Furthermore, at a concentration of 50 mg/mL, ZnO showed superior inhibitory capability against *B*. *subtilis* at all evaluated time points compared to the positive control. However, ZnO exhibited a lesser growth inhibitory effect on *V*. *parahaemolyticus*. Specifically, after 24 hours of treatment, ZnO at 5 mg/mL eradicated more than 50% of *V*. *parahaemolyticus*, while concentrations of 10 mg/mL and 50 mg/mL eliminated more than 61% and 78%, respectively. In contrast, the positive control tetracycline 0.003 mg/mL eradicated over 92% of *V*. *Parahaemolyticus* after 24 hours.

**Table 1:** Percentage of growth inhibitions between different conditions with the three bacteria *via* time points.

	*E*. *coli*				*B*. *subtilis*				*V*. *parahaemolyticus*			
	2h	4h	6h	24h	2h	4h	6h	24h	2h	4h	6h	24h
Tetracycline 0.003 mg/ml	89	91	93	98	90	93	93	96	85	98	98	92
ZnO 5 mg/ml	81	83	85	89	84	92	93	96	41	45	46	53
ZnO 10 mg/ml	85	86	88	91	86	92	94	96	47	55	56	61
ZnO 50 mg/ml	86	89	91	95	93	96	97	98	63	73	75	78

**Fig 4:**
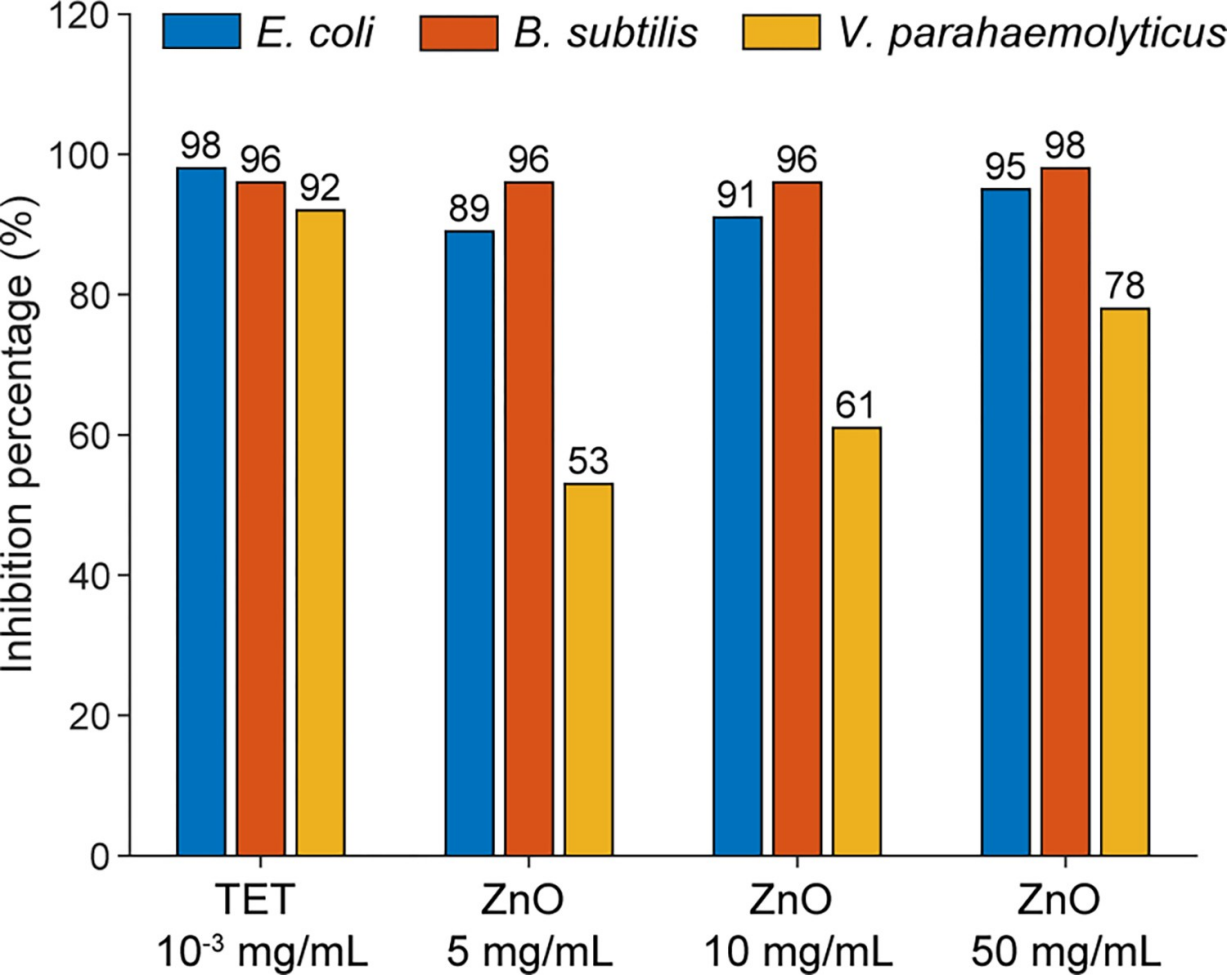
The inhibition percentage of each bacterium after 24 hours of treatment with 0.003 mg/mL TET and 5, 10, or 50 mg/mL ZnO NRs.

To better illustrate the bactericidal efficacy of different concentrations of ZnO, the bar plot of growth inhibition percentages has been reorganized and is displayed in Fig 4. Overall, ZnO nanorods demonstrate a strong potential to inhibit the growth of both Gram-positive and Gram-negative bacteria, such as *E*. *coli* and *B*. *subtilis*. Notably, they show significant inhibition against the *B*. *subtilis* strain, even at a concentration as low as 5 mg/mL.

**Fig 5:**
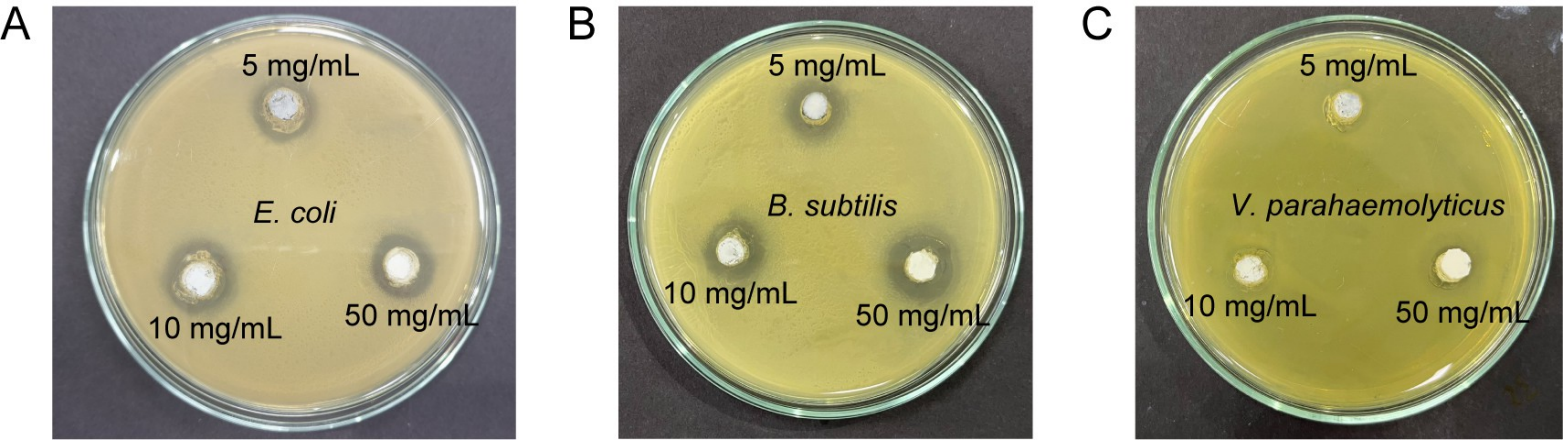
Images of the inhibition zones for three bacteria after 24 hours of growth at 37°C with different doses of ZnO treatments.

The antibacterial potential of ZnO nanorods was further demonstrated in the inhibition zone images of three types of bacteria cultured with ZnO for 24 hours (Fig 5). *E*. *coli* and *B*. *subtilis* exhibited similar diameters of inhibition zones at concentrations of 5 mg/ml and 50 mg/ml, measuring approximately 13.3 mm and 16 mm, respectively (Table 2). However, there was a noticeable difference in the size of the inhibition zone for ZnO nanorods at 10 mg/ml, with a diameter of 14 mm in *E*. *coli* and 15.3 mm in *B*. *subtilis*. In the case of *V*. *parahaemolyticus*, the inhibition zone was not visible. Still, there were approximate measurements of 10 mm, 11 mm, and 13 mm at concentrations of 5 mg/ml, 10 mg/ml, and 50 mg/ml, respectively. Table 2 also compares the effect of 0.003 mg/mL tetracycline on three bacteria. The antibacterial activities of different tetracycline concentrations on these three bacteria, as presented in the S2 File, indicate that none of the bacteria used in this study are antibiotic-resistant. Furthermore, the comparable measured inhibition of ZnO at 5 mg/mL and tetracycline at 0.003 mg/mL demonstrated the effectiveness of ZnO nanorods as an antibacterial agent against *Bacillus subtilis*, *E*. *coli*, and *V*. *parahaemolyticus*. Specifically, ZnO is most effective against *B*. *subtilis*, followed by *E*. *coli*, and least effective against *V*. *parahaemolyticus*.

**Table 2:** The diameters of inhibition zones of three bacteria with different dose treatments of ZnO and 0.003 mg/ mL TET after growth in 24 hours at 37°C.

	TET 0.003 mg/mL	ZnO 5 mg/mL	ZnO 10 mg/mL	ZnO 50 mg/mL
*E*. *coli*	12, 12, 13	13, 13, 14	13, 14, 15	15, 16, 17
*B*. *subtilis*	10, 10, 11	13, 13, 14	14, 15, 17	16, 16, 16
*V*. *parahaemolyticus*	13, 13, 14	10, 10, 10	11, 11, 11	13, 13, 14

**Fig 6:**
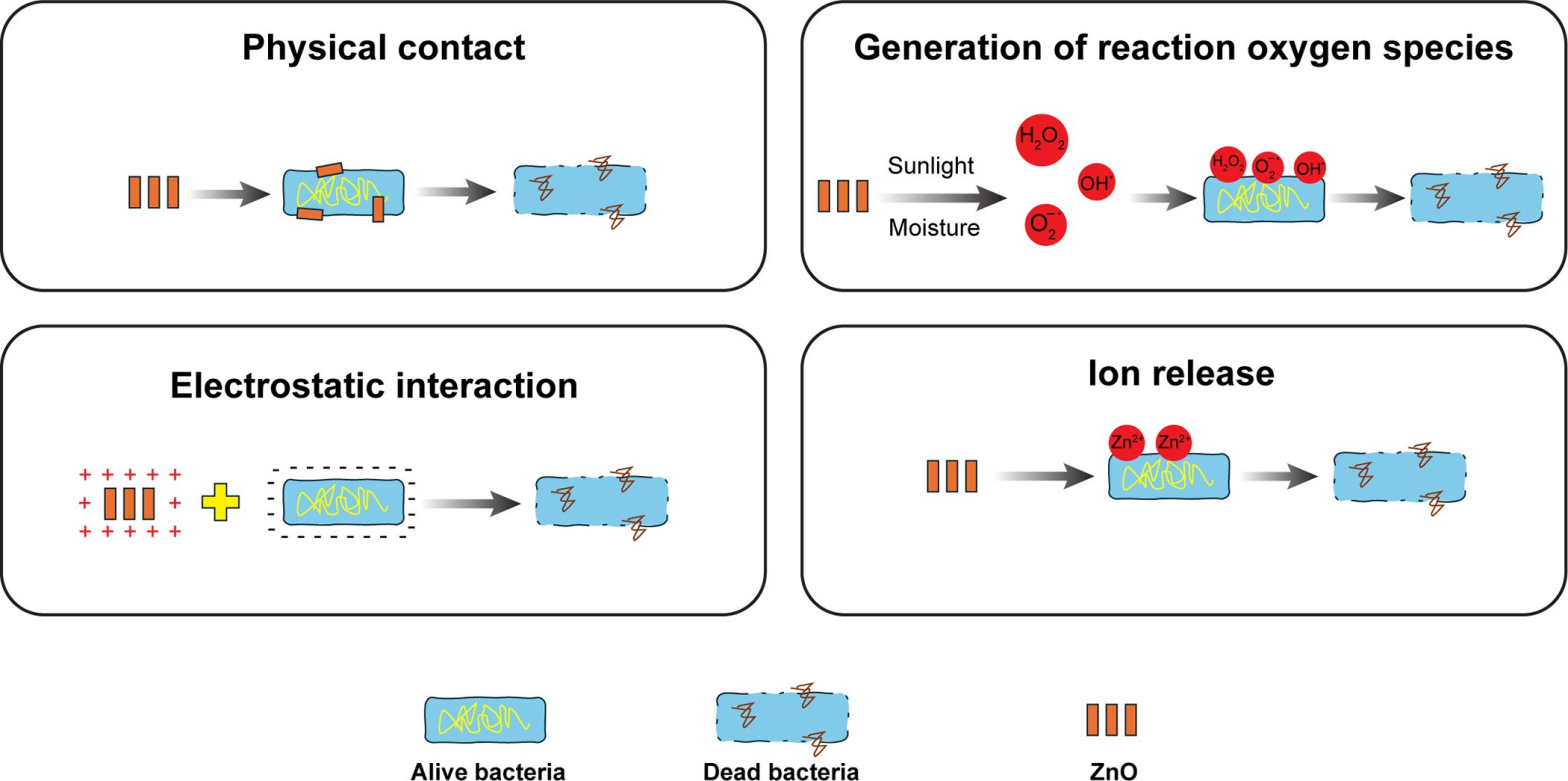
The hypothesis of the bactericidal mechanism of ZnO NRs on the bacterial.

The potent antibacterial properties of ZnO nanorods, as illustrated in Fig 6, can be attributed to several mechanisms, both contact and non-contact-based [25–27]. First, physical contact between ZnO nanorods and bacterial cells disrupts the cell membrane integrity through mechanical stress, leading to leakage of intracellular substances and potential cell death [28]. Second, under exposure to sunlight and humidity, ZnO nanorods generate reactive oxygen species (ROS) such as hydrogen peroxide (H₂O₂), hydroxyl radicals (OH^•^), and superoxide anions (O₂^−•^) [29]. These ROS induce oxidative stress, damaging vital cellular components like lipids, proteins, and DNA, which ultimately causes cell death [30]. Third, ZnO nanorods release Zn^2^⁺ ions, which disrupt essential cellular processes by interfering with enzyme activities and membrane integrity, culminating in bacterial death [31]. Finally, the positive charge on the surface of ZnO nanorods attracts negatively charged bacterial membranes, enhancing electrostatic interactions that disrupt the membrane and increase antibacterial effectiveness [32].

### Bactericidal efficacy of ZnO nanorods against *B*. *subtilis*

**Fig 7:**
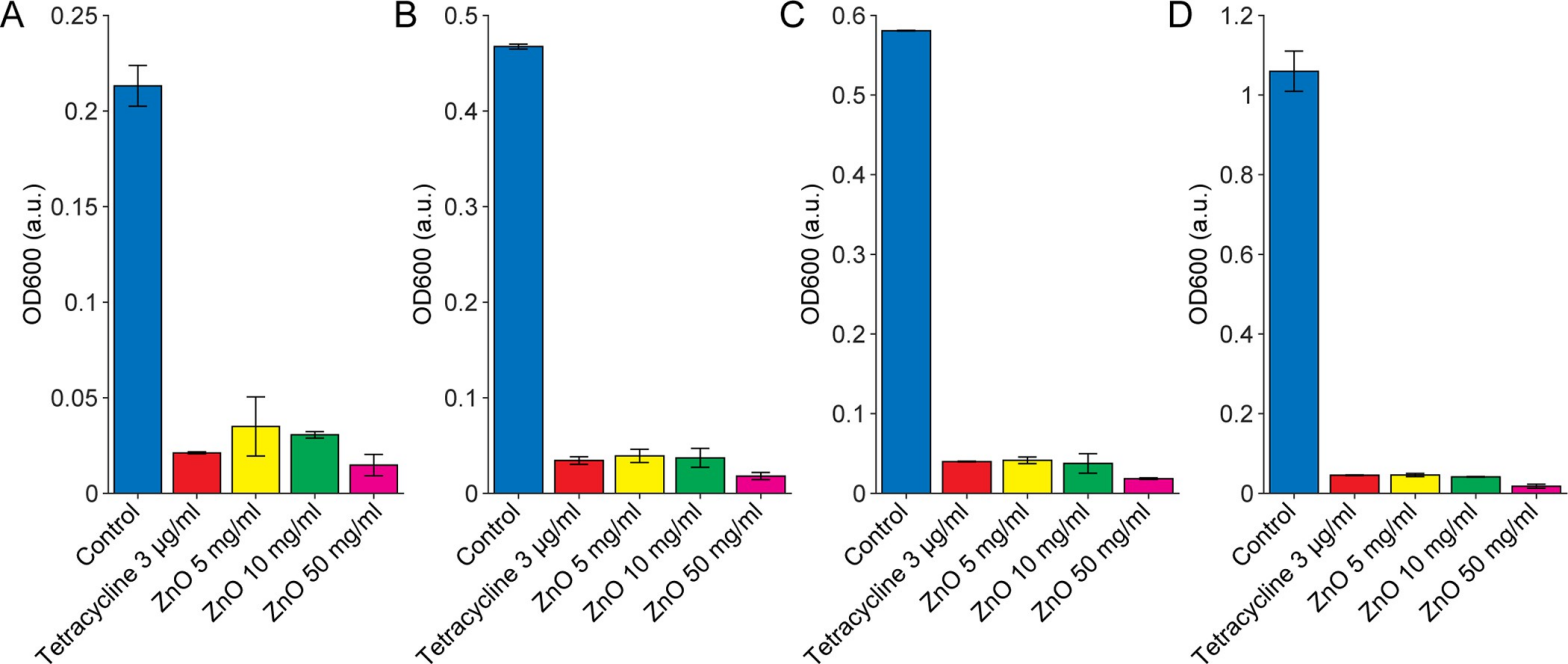
The antimicrobial activity of ZnO nanorods against *B*. *subtilis* was evaluated through OD 600 measurements taken at different time intervals. (A) 2 hours, (B) 4 hours, (C) 6 hours, and (D) 24 hours.

One aim of this study is to explore the bactericidal efficacy of ZnO NRs over time and to identify the optimal dose of ZnO that is comparable with antibiotics. We focus on *B*. *subtilis* as a representative example to establish the analysis. The bacterial growth, measured through OD_600_ after treatment with ZnO NRs and under negative and positive control, is monitored at different times, as shown in Fig 7.

All tested concentrations of ZnO (5, 10, 50 mg/ml), along with the positive control, tetracycline (0.003 mg/ml), effectively inhibited the proliferation of *B*. *subtilis* at 2, 4, and 6 hours. Significant differences were observed between all *B*. *subtilis* groups treated with either ZnO or tetracycline and the untreated control (Fig 7). Since the doubling time for all tested bacterial species is less than one hour, we began our analysis at the two-hour mark. After 2 hours, the bacteria were either in the lag phase or just starting the exponential phase, during which they adapt to their environment, synthesize necessary enzymes, and prepare for cell division [33]. Further analysis revealed that ZnO consistently inhibited *B*. *subtilis* growth by over 96% between 4 and 24 hours, performing as well as or better than tetracycline. The 50 mg/mL ZnO concentration exhibited the highest inhibitory effect at all time points. At 5 mg/mL, ZnO matched the inhibitory capability of tetracycline at 4, 6, and 24 hours, indicating that even a low concentration of ZnO is highly effective. These findings suggest that a 5 mg/mL concentration of ZnO could be an optimal choice for further detailed studies due to its efficacy comparable to that of tetracycline.

**Fig 8:**
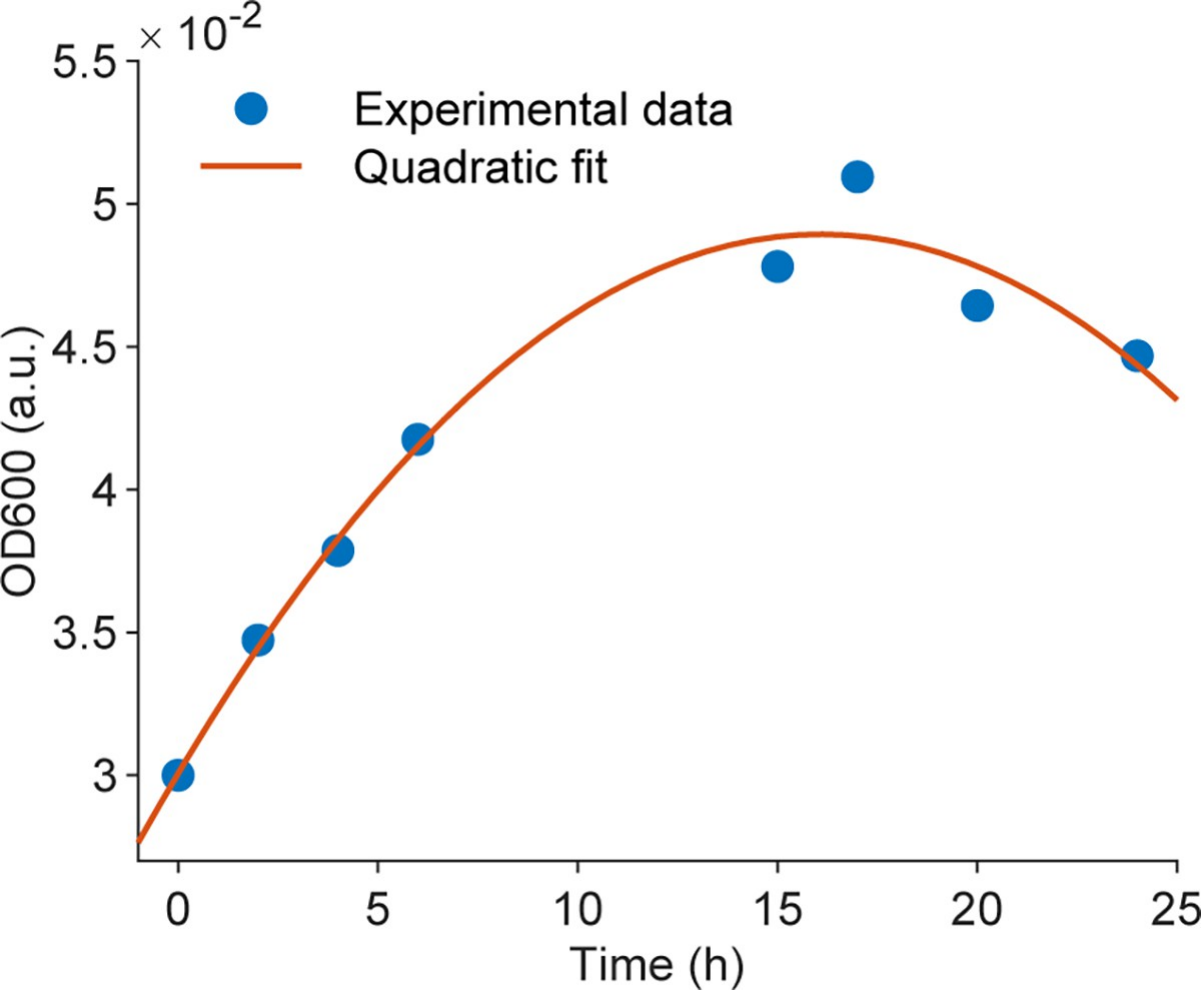
OD_600_ measurements of *B*. *subtilis* treated with 5 mg/mL ZnO NRs over time. Experimental data (blue dots) and quadratic fit of the experimental data (red line).

At an optimal concentration of 5 mg/mL, comparable to 0.003 mg/mL of TET, we further explored the analytical model of OD_600_ over time. We repeated the OD_600_ measurements for ZnO at 5 mg/mL with *B*. *subtilis* at intervals of 0, 2, 4, 6, 15, 17, 20, and 24 hours. Remarkably, the OD_600_ values at 2, 4, 6, and 24 hours were consistent with those reported in the S1 File, confirming the stable bactericidal properties of ZnO and the reliability of the results. Specifically, Fig 8 illustrates the changes in OD_600_ over 24 hours based on the experimental data. A quadratic regression model was developed to describe the effect of ZnO NRs at a concentration of 5 mg/mL on *B*. *subtilis*, which is expressed by the following equation:

$$y\;=\;7.295\times 10^{-5}\times x^{2}+0.002347\times x+0.03006,$$ (2)

where *y* is the OD_600_ of *B*. *subtilis* after being treated with 5 mg/mL ZnO NRs at time *x* (hour).

The model’s validity was further confirmed by measuring the optical density OD at 600 nm at different time points, including 1h, 3h, 5h, and 22 h (Table 3). The recovery percentage was calculated by $100\%\times \frac{Calculated\;{OD}_{600}}{Measured\;{OD}_{600}}$. The calculated OD_600_ values were determined by substituting the test time into Eq (2). Table 3 shows a strong correlation between the calculated and measured OD_600_ values, especially at extended durations following treatment with ZnO at 5 mg/mL, where the recovery percentages approached 100% in all tests. These results again confirm the stability of ZnO’s bactericidal properties and the fitting function’s reliability.

**Table 3:** Validation of a fitting model for OD_600_ of *B*. *subtilis* treated by ZnO 5 mg/mL.

Time (hour)	Measured OD_600_	Calculated OD_600_	Recovery (%)
1	0.0332	0.0323	97.32
3	0.0353	0.0364	103.32
5	0.0399	0.0400	100.19
22	0.0450	0.0464	103.06

**Fig 9:**
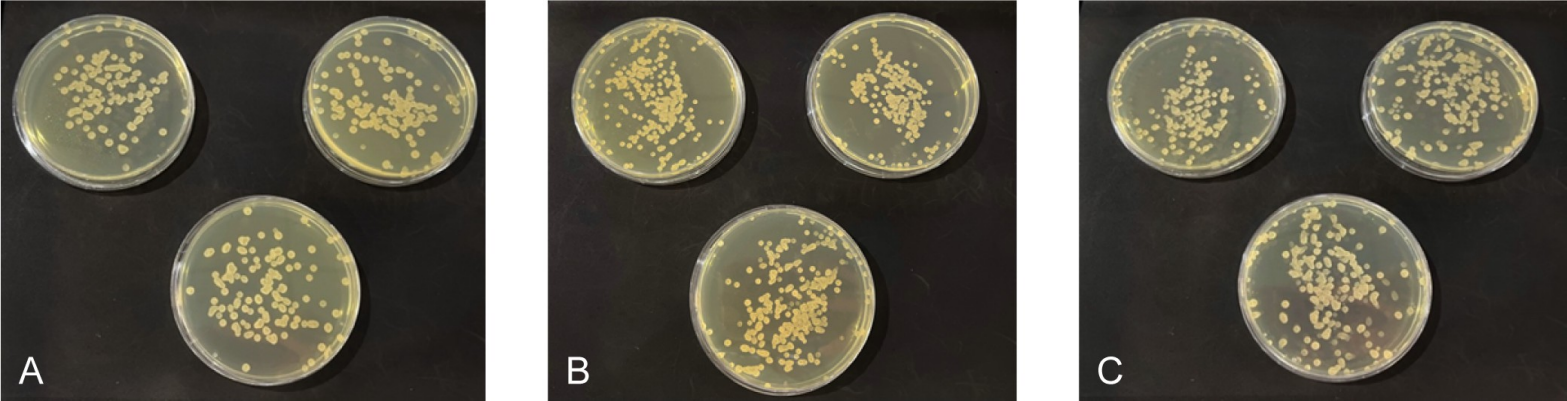
Culturing of *B*. *subtilis* bacteria after 2 hours. (A) without any treatment, serving as the negative control; (B) treated with 0.003 mg/mL of tetracycline (TET), used as the positive control; (C) treated with 5 mg/mL of ZnO.

To further evaluate the reliability of the antibacterial effects, we utilized culture techniques to quantify the bacterial count. Fig 9 presents data recorded 2 hours post-treatment. The colony count and the calculated number of bacteria (CFU/mL) from the original suspension are summarized in the S3 File. The CFU/mL count for *B*. *subtilis* is also detailed in Table 4. Based on these estimates, the bactericidal efficiency was calculated using Eq (3) and is presented in Table 4.


$$\%\;Bactericidal\;efficiency=\frac{{CFU}_{control}-{CFU}_{test}}{{CFU}_{control}}\times 100\%$$ (3)

**Table 4:** CFU and bactericidal efficiency following treatment of *B*. *subtilis* with 0.003 mg/L tetracycline and 5 mg/mL zinc oxide (ZnO) using the culturing method.

Time (hour)	Bacteria in the original suspension (CFU/mL)			Bactericidal efficiency (%)	
	Negative control	Positive control	ZnO	Positive control	ZnO
2	20.86×10^6^	3.45×10^6^	2.7×10^5^	83.46	98.70
4	26.92×10^6^	2.29×10^6^	2.78×10^5^	91.49	98.82
6	120×10^6^	3.14×10^6^	5.3×10^5^	97.38	99.56
24	52×10^8^	3.19×10^6^	9.46×10^6^	99.93	99.87

As illustrated in Table 4, within just 2 hours, 5 mg/mL of ZnO NRs eradicated 98.7% of the bacterial population, initially consisting of 4.26×10^6^ bacteria. In comparison, tetracycline initially showed a lower bactericidal efficiency of 83.46% at 2 hours but gradually increased to 91.49%, 97.38% at 4 and 6 hours, and reached 99.93% after 24 hours. By the 24-hour mark, ZnO exhibited a similar antibacterial efficiency to tetracycline, achieving approximately 99.9%. These findings confirm that ZnO has potent antibacterial effects on *Bacillus subtilis*, with nearly the same efficiency as the common antibiotic tetracycline at 0.003 mg/mL.

These remarkable results open the door for further exploration into the bactericidal properties of ZnO NRs on spiked samples and potential enhancement factors, such as UV light exposure or alterations in the pH of samples. This study builds upon previous work, as outlined in Table 5, by enhancing the efficacy of ZnO nanomaterials in killing pathogenic bacteria and simplifying the synthesis process. Firstly, ZnO nanorods have demonstrated higher efficacy than other forms, such as bulk ZnO or spherical nanoparticles. The explanation for this phenomenon might be that ZnO NRs have a high surface-to-volume ratio, which enhances their interaction with bacterial cells and can lead to more effective antibacterial action. Their specific surface structure and defect sites contribute to the generation of ROS, which are crucial for their antibacterial properties. Additionally, the rod-like shape of ZnO NRs allows them to penetrate bacterial cell walls more effectively, causing mechanical damage and cell lysis. Under UV light, ZnO NRs exhibit enhanced photocatalytic activity, generating ROS such as hydroxyl radicals and superoxide ions more efficiently than spherical nanoparticles. Furthermore, the surface chemistry of ZnO NRs, including their ability to adsorb onto bacterial cell walls or interact with microbial metabolic pathways, can influence their antibacterial efficacy. Overall, the unique morphology, surface characteristics, and interactions of ZnO NRs result in distinct antibacterial mechanisms compared to other nanoparticle forms. Secondly, in this work, ZnO NRs are synthesized at low temperatures within a short period (100°C for 5 hours), requiring minimal concentrations of materials (5 mg/mL) compared to tetracycline. Unlike other studies that require high temperatures for annealing or calcination, this method is more efficient and cost-effective (Table 5). Furthermore, this research has successfully developed and validated a predictive model to estimate bactericidal efficiency, providing a robust framework for future studies aimed at customizing ZnO-based treatments for various bacterial strains. Lastly, the bactericidal efficiency of ZnO NRs in this work was comparable to that of TET at 0.003 mg/mL.

**Table 5:** Comparison of this study to previous research: The minimum inhibitory concentration (MIC) of ZnO listed in the column pertains to the highlighted bacteria (*E*. *coli*, *B*. *subtilis*, or both).

ZnO morphology	Preparation method and condition	Bacterial	MIC	Ref
Nanoparticles	Probe sonication method: ultrasonic pulse, hydrothermal, and annealing at 500°C.	*E*. *coli*, *Pseudomonas aeruginosa*, *Klebsiella oxytoca*	1–5 mg/mL	[34]
Nanoparticles	Hydrothermal and calcination at 600°C for 4 h in a muffle furnace	*E*. *coli*, *Staphylococcus aureus*, *Pseudomonas aeruginosa*, *etc*.	25 μg/ml	[35]
Nanoparticles	Pulse microwave-assisted using microwave irradiation at 700 W at 60°C for 30 minutes and calcinated at 500°C.	*B*. *subtilis*	≥ 50 ppm	[36]
Nanoparticles	Co-precipitation method: 700°C for 3h in a muffle furnace	*E*. *coli*, *Proteus vulgaris*, *Staphylococcus aureus*, and *Streptococcus mutans*	10 mg/mL	[37]
Nanorods	Hydrothermal: 100°C, 5 hours	*E*. *coli*, *B*. *subtilis*	5 mg/mL	This work

In conclusion, the development of zinc oxide nanorods as antimicrobial agents presents a promising solution to the global challenge of antibiotic resistance. The unique bactericidal mechanisms of ZnO nanorods, including the generation of reactive oxygen species and membrane disruption, offer a multi-faceted approach to combating bacterial infections, making it significantly more difficult for bacteria to develop resistance. The results of this study demonstrate that ZnO nanorods exhibit potent antimicrobial activity at low concentrations, comparable to conventional antibiotics like tetracycline, suggesting their potential as a viable alternative or supplement to existing treatments. Moreover, the flexibility in engineering nanoparticle properties, such as size and shape, opens up the possibility for targeted and sustained antimicrobial action, reducing side effects and improving treatment precision. Beyond their immediate application as nano-antibiotics, ZnO nanoparticles could be incorporated into a variety of materials, including medical devices, wound dressings, and packaging, providing long-term antimicrobial protection in clinical, agricultural, and industrial settings.

Looking forward, the integration of ZnO nanorods into antimicrobial strategies could significantly reduce reliance on traditional antibiotics, slowing the spread of resistance. While the results of this study are highly promising, further research is required to fully explore the long-term safety and environmental impacts of ZnO nanoparticles. Their potential cytotoxicity, bioaccumulation, and effects on non-target microorganisms must be carefully assessed through comprehensive in vivo studies and environmental risk assessments. Additionally, regulatory frameworks will need to adapt to accommodate the novel nature of nanoparticle-based therapeutics to ensure their safe and effective use in clinical and industrial applications.

## Conclusions

This investigation into the use of zinc oxide nanorods as nano-antibiotic agents highlights their significant bactericidal potential against pathogens like *Escherichia coli*, *Bacillus subtilis*, and *Vibrio parahaemolyticus*, offering a promising alternative to traditional antibiotics compromised by increasing resistance. ZnO nanorods were synthesized using a low-temperature hydrothermal process in just 5 hours, a much shorter duration than previous research. These nanorods demonstrated comparable effectiveness to tetracycline at inhibiting bacterial growth, with an optimal concentration of 5 mg/mL, achieving up to 99.87% bacterial reduction within one day. The development and validation of a predictive model to estimate bactericidal efficiency further emphasize the robust potential of ZnO nanorods as a scalable and environmentally friendly antimicrobial solution. The findings suggest that ZnO nanorods, with their potent and efficient bactericidal properties, could play a crucial role in addressing the global challenge of antibiotic resistance, paving the way for new strategies in antimicrobial applications.

## Supporting information

S1 File OD_600_ measurements of the negative and positive control and samples treated with ZnO over time for three bacterial species. (PDF)

S2 File Inhibition zone experiments of various Tetracycline concentrations with the bacteria used in this study. (PDF)

S3 File The Colony count and calculated number of bacteria in the original suspension (CFU/mL). (PDF)

S1 Graphical abstract (TIF)
