# Supplementary material for: Single-step hydrothermal synthesis of zinc oxide nanorods for potential use as nano-antibiotics without seeding or bases
Source: PLoS One. 2024 Nov 4;19(11):e0313224. doi: 10.1371/journal.pone.0313224 (PMC11534225; doi:10.1371/journal.pone.0313224)
Supplement: S1 File — (PDF) [file pone.0313224.s001.pdf]

S1 Tables. OD_{600} measurements of the negative and positive control and samples treated with ZnO over time for three bacterial species. Table 1: *E. coli*, Table 2: *B. subtilis*, Table 3: *V. parahaemolyticus*

Table 1.

E. coli	**0h**	**2h**	**4h**	**6h**	**24h**
**Control**	0.03	0.151816	0.242235	0.321592	0.642554
**Tetracycline 0.003 mg/ml**	0.03	0.016211	0.020928	0.02156	0.01256
**ZnO 5 mg/ml**	0.03	0.029067	0.040494	0.048128	0.069088
**ZnO 10 mg/ml**	0.03	0.022928	0.033271	0.037278	0.057739
**ZnO 50 mg/ml**	0.03	0.02182	0.027636	0.029779	0.032107

Table 2.

B. subtilis	**0h**	**2h**	**4h**	**6h**	**24h**
**Control**	0.03	0.213161	0.467419	0.580774	1.059677
**Tetracycline 0.003 mg/ml**	0.03	0.021145	0.0343	0.039796	0.045471
**ZnO 5 mg/ml**	0.03	0.034977	0.039136	0.041455	0.046023
**ZnO 10 mg/ml**	0.03	0.030608	0.03713	0.037515	0.041384
**ZnO 50 mg/ml**	0.03	0.014741	0.018067	0.018436	0.017882

Table 3.

V. parahaemolyticus	**0h**	**2h**	**4h**	**6h**	**24h**
**Control**	0.03	0.09573255	0.13736417	0.1475778	0.16788898
**Tetracycline 0.003 mg/ml**	0.03	0.01476974	0.0032531	0.0032531	0.01404765
**ZnO 5 mg/ml**	0.03	0.0566	0.0754	0.07946667	0.07973333
**ZnO 10 mg/ml**	0.03	0.05056088	0.06164159	0.06533516	0.06590971
**ZnO 50 mg/ml**	0.03	0.03575851	0.03671053	0.03689628	0.03710526
