# Supplementary material for: Single-step hydrothermal synthesis of zinc oxide nanorods for potential use as nano-antibiotics without seeding or bases
Source: PLoS One. 2024 Nov 4;19(11):e0313224. doi: 10.1371/journal.pone.0313224 (PMC11534225; doi:10.1371/journal.pone.0313224)
Supplement: S2 File — (PDF) [file pone.0313224.s002.pdf]

S2 Fig. Inhibition zone experiments of various Tetracycline concentrations with the bacteria used in this study.

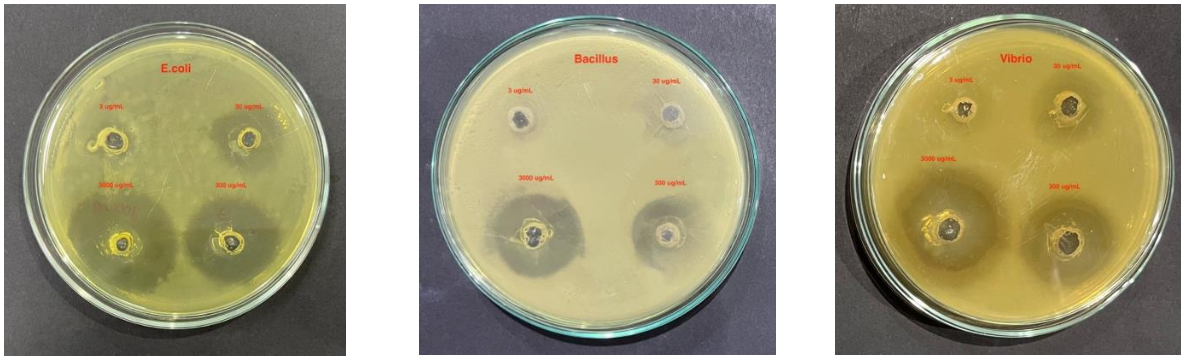

S2 Table. Inhibition zone experiments of various Tetracycline concentrations with the bacteria used in this study.

Tetracycline (µg/mL)	*B. subtilis*	*V. parahaemolyticus*	*E. coli*
3	10,10,11	13,13,14	12,12,13
30	14,15,15	18,19,20	19,19,20
300	20,20,20	23,24,25	25,26,26
3000	22,22,22	33,34,34	31,31,34
