# Supplementary material for: Single-step hydrothermal synthesis of zinc oxide nanorods for potential use as nano-antibiotics without seeding or bases
Source: PLoS One. 2024 Nov 4;19(11):e0313224. doi: 10.1371/journal.pone.0313224 (PMC11534225; doi:10.1371/journal.pone.0313224)
Supplement: S3 File — (PDF) [file pone.0313224.s003.pdf]

**S3 File. The Colony count and calculated number of bacteria in the original suspension (CFU/mL):** of *B. subtilis* as negative control (Table 1), Tetracycline 0.003 mg/mL as positive control (Table 2), and ZnO NRs 5 mg/mL (Table 3).

The number of bacteria N in the original suspension (CFU/mL) is calculated by:

$$N\left(\frac{CFU}{mL}\right) = \frac{Colony\ count_{Average} \times Dilution\ factor}{Volume\ (mL)}$$

**S3 Table. Table 1 of Culturing of *B. subtilis***

| Time (h) | Colony count | | | Volume (mL) | Dilution factor | Bacteria in the original suspension (CFU/ml) |
|---|---|---|---|---|---|---|
| | Plate 1 | Plate 2 | Plate 3 | | | |
| *B. Subtilis* (Control) | | | | 0.05 | | |
| 0 | 219 | 201 | 219 | 0.05 | 1:1000 | $4.26\times10^{6}$ |
| 2 | 107 | 102 | 104 | 0.05 | 1:10000 | $20.86\times10^{6}$ |
| 4 | 68 | 62 | 72 | 0.025 | 1:10000 | $26.92\ \times10^{6}$ |
| 6 | 59 | 49 | 73 | 0.05 | 1:100000 | $120\times10^{6}$ |
| 24 | 22 | 25 | 32 | 0.05 | 1:10000000 | $52\times10^{8}$ |

**S3 Table. Table 2 of Culturing of *B. subtilis* treated by Tetracycline 0.003 mg/mL**

| Time (h) | Colony count | | | Volume (mL) | Dilution factor | Bacteria in the original suspension (CFU/ml) |
|---|---|---|---|---|---|---|
| | Plate 1 | Plate 2 | Plate 3 | | | |
| *B. subtilis* (Tetracycline) | | | | 0.05 | | |
| 2 | 153 | 157 | 208 | 0.05 | 1:1000 | $3.45\times10^{6}$ |
| 4 | 126 | 110 | 108 | 0.05 | 1:1000 | $2.29\times10^{6}$ |
| 6 | 142 | 150 | 180 | 0.05 | 1:1000 | $3.14\times10^{6}$ |
| 24 | 160 | 178 | 141 | 0.05 | 1:1000 | $3.193\times10^{6}$ |

**S3 Table. Table 3 of Culturing of *B. subtilis* treated by ZnO NRs 5 mg/mL**

| Time (h) | Colony count | | | Volume (mL) | Dilution factor | Bacteria in the original suspension (CFU/ml) |
|---|---|---|---|---|---|---|
| | Plate 1 | Plate 2 | Plate 3 | | | |
| *B. subtilis* (ZnO) | | | | 0.05 | 0.00001 | |
| 2 | 131 | 140 | 136 | 0.05 | 1:100 | $2.7\times10^{5}$ |
| 4 | 144 | 140 | 134 | 0.05 | 1:100 | $2.78\times10^{5}$ |
| 6 | 30 | 29 | 21 | 0.05 | 1:1000 | $5.3\times10^{5}$ |
| 24 | 49 | 42 | 51 | 0.05 | 1:10000 | $9.46\times10^{6}$ |
