# Supplementary figures and images for: Single-step hydrothermal synthesis of zinc oxide nanorods for potential use as nano-antibiotics without seeding or bases
Source: PLoS One. 2024 Nov 4;19(11):e0313224. doi: 10.1371/journal.pone.0313224 (PMC11534225; doi:10.1371/journal.pone.0313224)

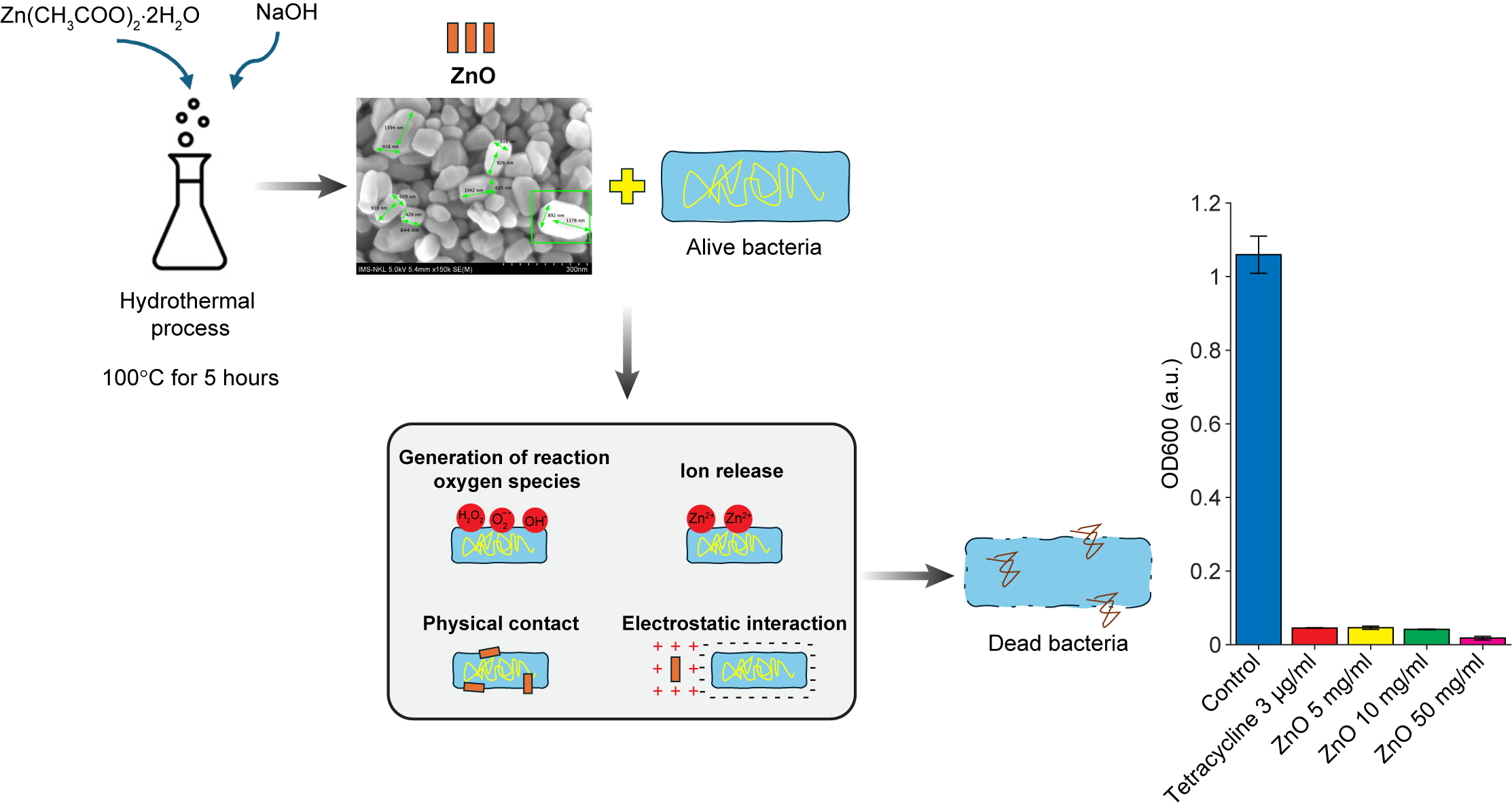

Supplement: S1 Graphical abstract — (TIF) [file pone.0313224.s004.tif]
